# A Critic Criticised

**Published:** 1860-10

**Authors:** 


### A CRITIC CRITICISED.

A HASTY reading of Dr. White's onslaught upon Taft's Operative Dentistry, in the September number of the Dental Cosmos, fetches up the adage, that those who live in glass houses, should not throw stones. I do not know how friend White may take it, but I feel called to say to him, in all kindness, that verbal criticism is a ticklish undertaking. He who criticises style, must do it in style, or provoke comparisons; and such "comparisons are odorous," always. Dr. White may be scientific, possibly; but probably he is not rhetorical—may be, not even grammatical; and so, it might have been more modest for him to have kept to the doctrine of Taft's book, and left the diction to speak for itself. Such is my feeble way of thinking; and I hope I may strengthen it by citations from Dr. White's article. Take, for instance, the beginning of the first paragraph:

"For want of space, we were obliged to defer further reference to this work in our last issue: nor would we do so now," etc.

Do what? Do further reference? Let us see: "nor would we do further reference now." Scarcely. Well, then, does "do" stand for "defer?" Give it a fair trial: "nor would we defer so now." Nonsense. Do what, Dr. White? Do tell!

The second paragraph opens as follows:

"The principle in the article on *Irregularity* has been carefully observed that, if we can not teach properly, it is better not to teach at all."

As a model of "perfect looseness," the sentence is all that heart could desire. With careful study, the fragments of sense may be gathered up, and stuck together thus:

"In the article on *Irregularity*, the principle that, if we can not teach properly, it is better not to teach at all, has been carefully observed."

I guess that is what the Doctor meant to say. But the next sentence can not be guessed into shape; the deformity is inherent and essential:

"If irregularity of the teeth is not a part of operative dentistry, it ought not to have been referred to in the work."

Now, it may be kind to inform critical editor of Cosmos (Dental as aforesaid) that "irregularity of the teeth is not a part of operative dentistry," nor, indeed, of any other branch of dentistry; though treatment of irregularity is. Perhaps "irregularity of the teeth is not a part of" the mouth; but it is that, if it is a part at all.

Omitting minor errors in this paragraph, such as a misuse of the subjunctive mood, a repetition of the phrase "most important" three times in six lines, a misapplication of prepositions, and the like, let us pause upon the following sublime grammar:

"To have referred to it at all by the author, it placed him under obligations to do so properly."

Is comment necessary? Not to readers of the Register, certainly; but, since I am writing mainly for the instruction of Dr. White, it is well to remark to him that the language, to be made at all passable, must be changed thus much, at least:

"For the author to have referred to it at all, placed him under obligations to have done so properly."

But our critic's rhetoric is worse than his grammar, if possible. For example, he speaks of "the old and false doctrine advanced as the principal cause of irregularity." A *doctrine* the *cause* of irregularity! Go to.

In the fourth paragraph, we find "another physiological fact," brought out in our italics as follows:

"The disproportion between the size of the *teeth* and the *jaws*, during the period of *their* eruption, is in many cases very great."

But could the Doctor inform us what is the period of the jaws' eruption?

Yet again, in the same paragraph, science is thus enriched:

"Teeth which are irregular during that time, usually correct themselves, when this disproportion exists; but when one tooth is in or outside of the arch, it [the tooth?] is another thing, and in such cases it is not always for want of room," [that it is another thing?]

But, as already intimated, our critic is keen upon words. Hear him:

"The author says that 'the teeth most liable to be out of position are the cuspidate,' (*cuspidati* is doubtless meant.")

"*Cuspidati is* doubtless meant; and so, when one says good night, *bonam noctem* "is doubtless meant;" but it is only a pedantic fool that expresses himself in such a manner. *Cuspidate* is a legitimate English adjective, and, in the construc tion referred to, there is an ellipsis after it of the word *teeth.* Dr. Taft deserves praise for having thus always preferred native terms to foreign ones, wherever the idea could be conveyed just as well. It is a mark of sound sense and of sound scholarship.

Furthermore, critic attempts to be pointed with two exclamation-points, quoting from Taft in this wise:

"In irregular teeth, parts are approximated, that nature did not intend should be brought together [! !]"

We are not clear what point critic may be aiming at, but presume he means to insist that nature intends all irregularities that exist. But he forgets that the word *nature,* without a qualifier, implies nature without a perversion.

Critic goes on to inform us that he has "not made a relative statistic" Well, we trust that, when he does make one, he will favor friends with a sight of it.

But see here again how he criticises rhetoric:

"We have seldom observed a series of reasons advanced, accounting for or explaining an affection, so indefinite and jumbled-up, as are contained under this heading."

Notice the rare elegance of that sentence—only it has "an affection so indefinite and jumbled-up," as to be utterly incurable.

But, speaking of affection, witness how our rhetorical critic sets a mouse-trap for the phrase, "death of the part affected," by asserting that, "when a part is dead, it ceases to be affected." Now, for the conclusion of this grammar lesson to-day, we inform him that *affected* is the perfect participle of the verb *affect;* that the perfect participle implies past time; and that therefore "part affected" signifies "part that was affected;" but that, when a thing is dead, it does not cease to be affected: it *has ceased* to be affected—except as a dead thing.

Finally, we may say of J. D. W.'s criticism, in J. D. W.'s own language, that "there are a good many interesting ideas contained in the article above referred to,"—interesting for the manner in which they are expressed; and we confidently count on gratitude of Cosmos for having called attention to them. Cosmos is under no obligations, however; our labor is a labor of love, and we shall take pleasure in rendering any future services that its necessities may seem to require.

C.

---

*Popular Medical Jurisprudence.*—The following verdict of a coroner's jury is said to have been recently rendered:

"We find that Mr. Fink died of a natural death produced by dyspepsy, owing to clams for dinner, and cholera morbus in the evening."

It had better read—*clams for dinner, and cholera morbus for supper!*

